# An Intelligent System to Sense Textual Cues for Location Assistance in Autonomous Vehicles

**DOI:** 10.3390/s23094537

**Published:** 2023-05-06

**Authors:** Salahuddin Unar, Yining Su, Pengbo Liu, Lin Teng, Yafei Wang, Xianping Fu

**Affiliations:** School of Information Science and Technology, Dalian Maritime University, Dalian 116026, China

**Keywords:** sensing system, autonomous vehicles, advanced driver assistance system, image sensing, vision sensor

## Abstract

The current technological world is growing rapidly and each aspect of life is being transformed toward automation for human comfort and reliability. With autonomous vehicle technology, the communication gap between the driver and the traditional vehicle is being reduced through multiple technologies and methods. In this regard, state-of-the-art methods have proposed several approaches for advanced driver assistance systems (ADAS) to meet the requirement of a level-5 autonomous vehicle. Consequently, this work explores the role of textual cues present in the outer environment for finding the desired locations and assisting the driver where to stop. Firstly, the driver inputs the keywords of the desired location to assist the proposed system. Secondly, the system will start sensing the textual cues present in the outer environment through natural language processing techniques. Thirdly, the system keeps matching the similar keywords input by the driver and the outer environment using similarity learning. Whenever the system finds a location having any similar keyword in the outer environment, the system informs the driver, slows down, and applies the brake to stop. The experimental results on four benchmark datasets show the efficiency and accuracy of the proposed system for finding the desired locations by sensing textual cues in autonomous vehicles.

## 1. Introduction

Autonomous vehicles (AV) have gained significant popularity in recent years due to the vast revolution in modern transportation systems. An autonomous vehicle is a self-driving vehicle that is efficient at perceiving its outer environment and moving without or with very limited human involvement. The various renowned reports and surveys predict that by 2030, autonomous vehicles will be capable and reliable enough to replace maximum human driving [1,2]. In this scenario, many new methods are being proposed to facilitate autonomous vehicles’ vision perception, sensing the outer environment, safety aspects, traffic laws and regulations, accident liability, and maintaining the surrounding map [3,4,5].

An autonomous vehicle can rely on multiple sensors, complex algorithms, actuators, machine learning tools, computer vision techniques, and reliable processors to take effect [6,7]. The autonomous vehicle perceives the outer environment with the help of numerous sensors and makes the decision by perceiving with the assistance of computer vision [8,9]. Each sensor’s configuration and mechanism varies, as an example in [10], the sideslip angle estimation algorithm for autonomous vehicles is proposed. The algorithm is based on a consensus Kalman filter that fuses measurements from a reduced inertial navigation system (R-INS), a global navigation satellite system (GNSS), and a linear vehicle-dynamic-based sideslip estimator.

Since the last few decades, advanced driver assistance systems (ADAS) are equally appreciated to avoid traffic accidents and to improve driving comfort in autonomous vehicles [11]. The ADAS systems are safe and secure systems designed to decrease the human error rate. These systems assist the driver through the advanced technologies to drive safely and thus, improve the driving performance. Several state-of-the-art methods have employed the inertial measurement unit (IMU) and a global navigation satellite system (GNSS) for vehicle localization. In [12], the authors have proposed a method for estimating the sideslip angle and attitude of an automated vehicle using IMU and GNSS. The method is designed to be robust against the effects of vehicle parameters, road friction, and low-sample-rate GNSS measurements. In [13], the method is proposed for estimating the yaw misalignment of an IMU mounted on a vehicle.

The ADAS systems utilize a combination of multiple sensors to perceive the outer environment and then either offers useful information to the driver or take some necessary actions such as applying the brake, changing the lane, turning left/right, etc. These systems are very helpful to decrease traffic congestion and smoothing traffic movement [14,15]. In the last three decades, multiple features of ADAS systems have been proposed, including cruise control, antilock braking system, auto-parking, power steering, lane centering, collision warnings, and others [16,17,18]. In autonomous vehicles, cameras are generally used as vision sensors. The vision-based ADAS utilizes multiple cameras to capture the images, analyze them, and take the appropriate actions whenever needed.

In state-of-the-art methods, multiple advanced features of the ADAS system are proposed. In [19], Liu et al. proposed a framework using SVM-based trail detection to achieve trail directions and tracking in a real-time environment. The vision-based framework is capable to detect and track the trails as well as scene understanding using a quadrotor UAV operator. Yang et al. [20] proposed two frameworks that show how the CNNs perceive and process the driving scenes with distinguishing visual regions. Gao et al. [21] proposed a method for a 3D surround view for ADAS that covers automobiles around the vehicle. The method helps the driver to be aware of the outer environment.

Liu et al. [22] presented a novel algorithm for detecting tassels in maize using UAV-based RGB imagery. The algorithm named YOLOv5-tassel, is based on the YOLOv5 object detection framework and incorporates several modifications to improve its performance on tassel detection. The authors included the modifications of a bidirectional feature pyramid network to effectively fuse cross-scale features, introduced a robust attention module to extract the features of interest before each detection head, and added an additional detection head to improve small-size tassel detection. Xia et al. [23] proposed a novel data acquisition and analytics platform for vehicle trajectory extraction, reconstruction, and evaluation based on connected automated vehicle (CAV) cooperative perception. The platform is designed to be holistic and capable of processing sensor data from multiple CAVs.

Wang et al. [24] proposed a lane-changing model for making decisions to either change the lane or produce trajectories. The model analyzes the vehicle kinematics of different states, their distances, and comfort level requirements. Chen et al. [25] proposed an instructor-like assistance system in order to avoid collision risk. The driver and the assistance system both assure the recommendation to control the vehicle. Gilbert et al. [26] proposed an efficient decision-making model which selects the least possible collision for AV. The model combines vehicle dynamics and maneuver trajectory paths to produce simulation results and multi-attribute decision-making techniques. Gao et al. [27] proposed a new vehicle localization system that is based on vehicle chassis sensors and considers vehicle lateral velocity. The system is designed to improve the accuracy of vehicle stand-alone localization in highly dynamic driving conditions during GNSS outages.

Xia et al. [10] presented a new algorithm for estimating the sideslip angle of an autonomous vehicle. The algorithm uses consensus and vehicle kinematics/dynamics synthesis to enhance the accuracy of the estimation under normal driving conditions. The proposed algorithm uses a velocity-based Kalman filter to estimate the errors of the reduced inertial navigation system (R-INS) and a consensus Kalman information filter to estimate the heading error. The consensus framework combines a novel heading error measurement from a linear vehicle-dynamic-based sideslip estimator with the heading error from the global navigation satellite system (GNSS) course. Liu et al. [28] proposed a novel kinematic-model-based vehicle slip angle (VSA) estimation method that fuses information from a GNSS and an IMU. The method is designed to be robust against the effects of vehicle roll and pitch, a low sampling rate of GNSS, and GNSS signal delay.

Since the early days of mechanical vehicles, safety has been one of the key concerns in automotive systems. Several attempts have been made to address safety concerns by developing safe and secure systems to protect the driver as well as prevent injuring pedestrians [29,30]. It is one of the safety aspects of an autonomous vehicle when the driver is preoccupied with searching for the desired location to stop. With our proposed system, the safety of AV can be increased drastically since the AV will automatically realize the desired locations in its surrounding. Rather than continually searching for the desired locations, our proposed system will automatically realize the textual cues present in the outer environment and suggest the driver to stop.

While driving on the road, AV performs multiple operations such as lane change, lane keeping, overtaking, and following the traffic rules. Several studies proposed and developed numerous methods for ADAS systems [31,32]. It is equally important for an autonomous vehicle to be aware of the textual cues appearing in its outer environment to take some decision or assist the driver, either to stop or drive. Thus, the key concern of this paper is to reduce human intervention in an autonomous vehicles.

In this paper, we propose a novel intelligent system based on the driver’s instruction for finding the desired locations using textual cues present in the outer environment for advanced driver assistance systems. For this, we combine computer vision and natural language processing (NLP) techniques to perceive textual cues. Computer vision methods train the system to interpret and perceive the visual world around the autonomous vehicle and NLP techniques emphasize the system with the ability to read, recognize, and derive the meaning from textual cues appearing in front of an autonomous vehicle. The key contributions of this paper are as follows:

- A novel intelligent system is proposed for AVs to find unsupervised locations.
- The proposed system is capable of sensing the textual cues that appear in the outer environment for determining desired locations.
- The proposed system is a novel development in the list of ADAS features of an autonomous vehicle.
- With the proposed system, the driver’s efforts for finding the desired locations will drastically be decreased.

The remainder of the paper is organized as follows. In Section 2, we describe the proposed system for finding the desired locations with textual cues and their formation as keywords. In Section 3, the experimental results are defined to show the efficiency and accuracy of the proposed system. Section 4 concludes the proposed work and presents the future directions.

## 2. Proposed System

**Figure 1:**
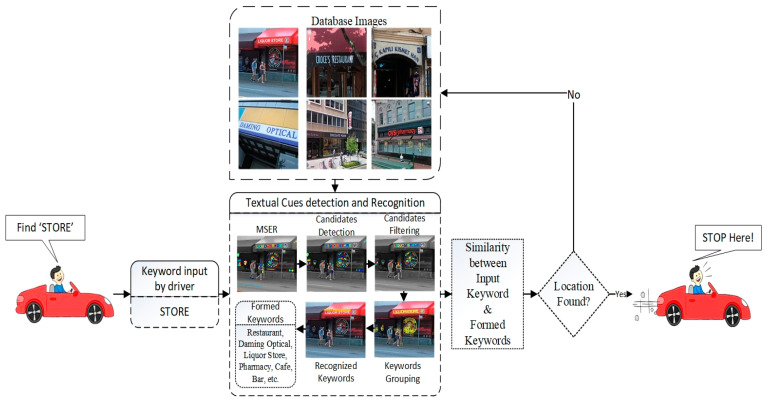
Schematic diagram of the proposed system.

In this section, we propose a novel intelligent system to find the desired locations using textual cues for an autonomous vehicle. Firstly, the driver inputs one or more keywords to the proposed system to find the desired locations. Secondly, the proposed system detects and localizes the textual cues appearing in the outer environment. The system will generate the keywords localized from the outer environment with detection and recognition methods. Finally, the system will execute similarity learning to find the similarity between the input keywords and the localized keywords from outer environment images. The schematic diagram of the proposed intelligent system is shown in Figure 1.

### 2.1. Textual Cues Detection

In order to detect, localize, and form the keywords from the outer environment, we employ text detection and localization technique. Firstly, we use affine transformation to deal with global distortion appearing within an input image and to improve the accuracy of the text to a more horizontal text. It takes an input image $I_{i}\in {\mathbb{R}}^{C_{i}\times H_{i}\times W_{i}}$ with channel $C_{i}$, height $H_{i}$, and width $W_{i}$ to produce an output image $I_{g}$. The affine transformation based on the arguments between the input image $I_{i}$ and output image $I_{g}$ is given as:

(1) $$\left[\begin{array}{l} x_{i}\\ y_{i} \end{array}\right]=T_{\theta }\left[\begin{array}{l} x_{g}\\ y_{g}\\ 1 \end{array}\right]$$

where $\left(x_{i},y_{i}\right)$ are the source coordinates of the input image and $\left(x_{g},y_{g}\right)$ are the required coordinates for the output image $I_{g}$. The output image $I_{g}$ is further rectified from the input image $I_{i}$ using bilinear interpolation, given as:

(2) $$I_{g}(x_{g},y_{g})=\sum_{n}^{H_{i}}\sum_{m}^{W_{i}}I_{i(n,m)}\max\left(0,1-\left|x_{i}-n\right|\right)\max\left(0,1-\left|y_{i}-m\right|\right)$$

where $I_{g}(x_{g},y_{g})$ is the pixel value of the rectified image $I_{g}$ at the location $\left(x_{g},y_{g}\right)$ and $I_{i(n,m)}$ is the pixel value of the input image $I_{i}$ at the location (n,m).

#### 2.1.1. Textual Candidates Detection

**Figure 2:**
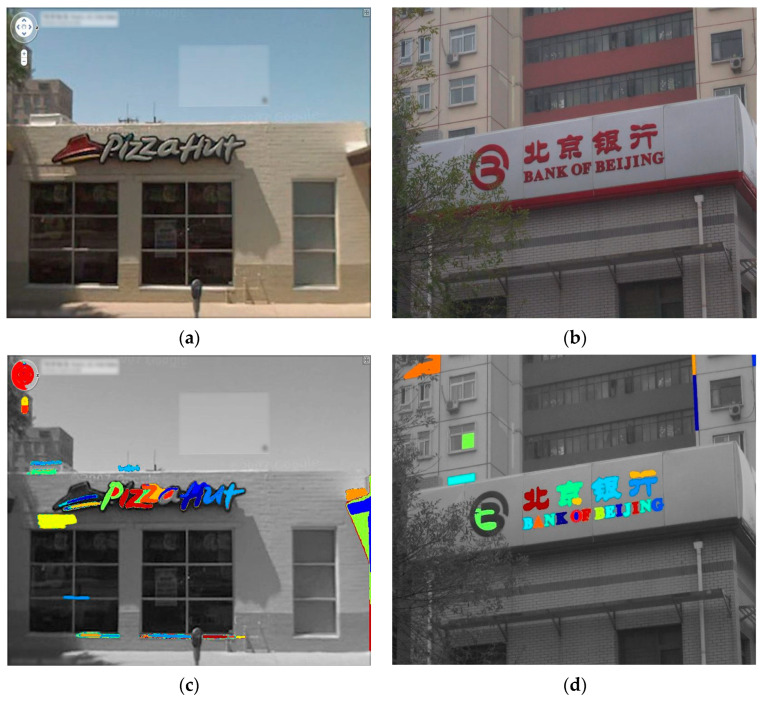
Textual candidates detection. (**a**,**b**) Original images. (**c**,**d**) Detected extremal regions.

The textual candidates detection aims to extract the position of textual regions in the outer environment. Since the text appearing in the outer environment generally has diverse contrast to its relative background and uniform color intensity, the maximally stable extremal region (MSER) technique is the best approach as it is widely used and considered the best region detector [33]. In order to detect the textual candidates appearing in the outer environment, we adopt the MSER approach for finding the corresponding candidates within the input image $I_{g}(x_{g},y_{g})$. For finding the extremal regions in the input image, the intensity difference is given as:

(3) $$\nu (R_{x})=\frac{\left|R(+\Delta )-R\right|}{\left|R\right|}$$

where |R| represents the extracted extremal regions area, R(+Δ) represents the extremal regions, +Δ specifies the increment of each extremal region R, and |R(+Δ)−R| shows the area difference between the two regions’ area. After applying the region detector, the obtained extremal regions are shown in Figure 2.

#### 2.1.2. Textual Candidates Filtering

The textual regions detected in the previous step using the MSER technique are further refined and rectified. First, we validate the size and the aspect ratio using geometric properties for textual candidates filtering, which is given as:

$$h_{\min}\leq h\leq h_{\max}$$

$$r_{\min}\leq h/w\leq r_{\max}$$

where *h* and *w* are the height and width of the aligned bounding box of segmented axes, respectively, and $h_{\min}$, $h_{\max}$, $r_{\min}$ and $r_{\max}$ are components to finetune.

The input image $(x_{d},y_{d})\in \ell_{d}$ having the size W×H and the predicted categorized result α and $\alpha^{\prime }$ with uncertain probability sequence p and $p^{\prime }$ is given as:

(4) $$\Theta_{t}(x_{d})=(\alpha ,p)$$

(5) $$\Theta_{s}(x_{d})=(\alpha^{\prime },p^{\prime })$$

where

(6) $$\alpha =(\alpha_{1},\alpha_{2},\alpha_{3},\ldots ,\alpha_{D})$$

(7) $$p=(p_{1},p_{2},p_{3},\ldots ,p_{D})$$

(8) $$\alpha^{\prime }=({\alpha^{\prime }}_{1},{\alpha^{\prime }}_{2},{\alpha^{\prime }}_{3},\ldots ,{\alpha^{\prime }}_{K})$$

(9) $$p^{\prime }=({p^{\prime }}_{1},{p^{\prime }}_{2},{p^{\prime }}_{3},\ldots ,{p^{\prime }}_{K})$$

where *D* and *K* represent the character sequence length. 

The input vector V is combined using the following properties:

$$\min(p_{1},p_{2},p_{3},\ldots ,p_{D})$$

$$\min({p^{\prime }}_{1},{p^{\prime }}_{2},{p^{\prime }}_{3},\ldots ,{p^{\prime }}_{K})$$

$$\mathrm{mean}(p_{1},p_{2},p_{3},\ldots ,p_{D})$$

$$\mathrm{mean}({p^{\prime }}_{1},{p^{\prime }}_{2},{p^{\prime }}_{3},\ldots ,{p^{\prime }}_{K})$$

The above four properties are probability characteristics in which mean represents the overall confidence score and min represents the least likely character.

Furthermore,

(10) $$D^{\prime }=\frac{D}{\theta }$$

(11) $$K^{\prime }=\frac{K}{\theta }$$

where θ is the constant parameter. The above two properties are used to normalize the number of characters between 0 and 1, and the following two properties are used for character width calculated as per geometric properties:

(12) $$\kappa =\frac{W}{D}$$

(13) $$\kappa^{\prime }=\frac{W}{K}$$

where κ represents the character width.

**Figure 3:**
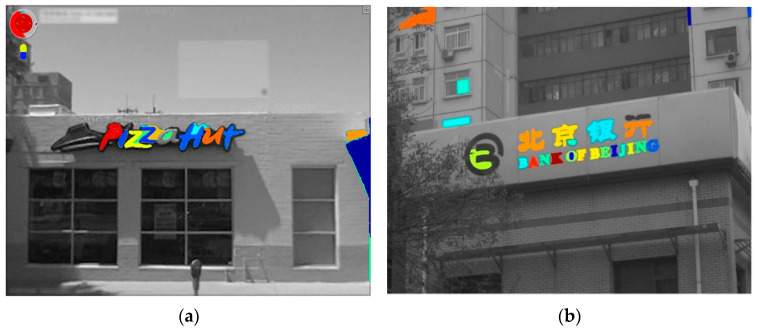
Textual candidates filtering. (**a**,**b**) non-textual objects filtered.

The localized regions which satisfy the above properties are then further processed and the remaining regions are discarded, as shown in Figure 3. The obtained localized regions consist of non-textual regions and may produce a false result for recognition. We further segment textual regions with the stroke responses of each image pixel. The corner points are used as the edges of two strokes. The corner points and stroke points establish the distortion of strokes. For this, we follow the corner detection approach [34], which applies the following selection criteria. Firstly, the matrix *M* for each pixel is calculated as follows:

(14) $$M=\sum_{\left(x,y\right)}w(x,y)\left[\begin{array}{cc} I_{x}^{2} & I_{x}I_{y}\\ I_{x}I_{y} & I_{y}^{2} \end{array}\right]$$

where w(x,y) represents the weight at position (x,y) for window center, $I_{x}$ and $I_{y}$ denotes the gradient value of pixel at position (x,y). The eigenvalues $\lambda_{1}$ and $\lambda_{2}$ of M matrix are calculated as:

(15) $$R=\min(\lambda_{1},\lambda_{2})$$

To compute the turning point of outer stroke endpoints, we use the following equation [35]:

(16) $$x^{*},y^{*}=\underset{x,y}{\arg\max}\left(\frac{(X_{2}-X_{1})\times y+(Y_{2}-Y_{1})\times x+X_{2}Y_{1}-X_{1}Y_{2}}{{\left(X_{2}-X_{1}\right)}^{2}+{\left(Y_{2}-Y_{1}\right)}^{2}}\right)$$

where $X_{1}$, $X_{2}$, $Y_{1}$, and $Y_{2}$ are the coordinates of the endpoints of the strokes; $x^{*}$ and $y^{*}$ are coordinates of the outermost points; and x,y denotes coordinates of every single point at the curve. The following equation is given to determine outer stroke points:

(17) $$P(x_{out},y_{out})=2P(x^{*},y^{*})-\frac{1}{2}(P(X_{1},Y_{1})+P(X_{2},Y_{2}))$$

where P(x,y) denotes a single point at the *x*-axis and *y*-axis in word image.

**Figure 4:**
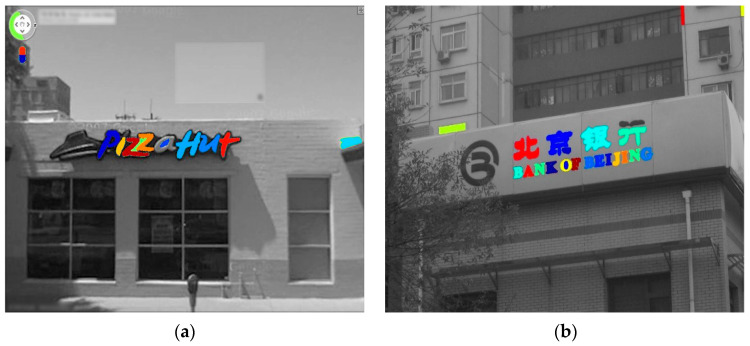
Optimal textual candidates. (**a**,**b**) Filtered textual candidates.

Given the corner point $P(X_{c},Y_{c})$ along with its adjacent corner $P(X_{n},Y_{n})$, the height $h_{area}$ and width $w_{area}$ of a moving window is determined as:

(18) $$h_{area}=2\alpha \times \left|Y_{c}-Y_{n}\right|$$

(19) $$w_{area}=2\alpha \times \left|X_{c}-X_{n}\right|$$

where α is a coefficient to normalize the area of the moving region among the corner points and is set between 0 and 1. Moreover, the moving area of outer strokes $P(X_{o},Y_{o})$ for the side length area $s_{area}$ is given as:

(20) $$s_{area}=\beta \times \left(\frac{(X_{2}-X_{1})\times Y_{o}+(Y_{2}-Y_{1})\times X_{o}+X_{2}Y_{1}-X_{1}Y_{2}}{{\left(X_{2}-X_{1}\right)}^{2}+{\left(Y_{2}-Y_{1}\right)}^{2}}\right)$$

where β is a coefficient to normalize the moving regions among the outer strokes and is set between 0 and 1. The final filtered localized textual regions are shown in Figure 4.

#### 2.1.3. Keywords Grouping and Recognition

The localized textual regions in the previous steps consist of individual text characters. In order to recognize and understand the meaning of these textual regions, these individual characters must be combined into text lines. This way, the localized textual regions may represent more meaningful information about the outer environment as compared to the individual characters. For example, the localized textual region consists of the “SCHOOL” versus the individual character set {C,O,L,O,S,H} where its meaning is lost due to the unordered sequence of the word [36,37,38].

In order to form the ordered keywords, we employ the grouping approach [39]. The key idea is to apply a rectangle $\omega s_{p}\times hs_{p}$ for each connected region having the center $\left(x_{p},y_{p}\right)$ and orientation $\theta_{p}$. Each associated region is considered to be a keyword candidate. The initial candidate regions having $w_{1}=0.4$ are refined to be the keyword with the following properties:

(1) The two adjacent textual candidates are associated with a new $w_{i}$ value.
(2) The achieved keyword candidate which is the combination of two candidates is obtained with curvilinear.

**Figure 5:**
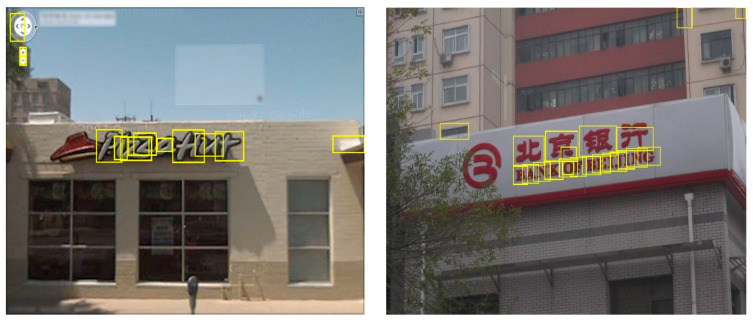
Keywords grouping using yellow bounding boxes.

If the centers of connected regions in τ are estimated normally with a *k*th order polynomial, then the candidate keyword τ(⊂ζ) is determined as curvilinear:

(21) $$\sqrt{\underset{f}{\min}\sum_{c_{p}\in \tau }\frac{1}{\left|\tau \right|}{\left(\left({y^{\prime }}_{p}-f({x^{\prime }}_{p}\right)\right)}^{2}}\leq \frac{\bar{s}}{4}$$

where $\left({x^{\prime }}_{p},{y^{\prime }}_{p}\right)$ is the rotated point of $\left(x_{p},y_{p}\right)$ and $\bar{s}=\frac{1}{\tau }\sum s_{p}$ is the average score. The bounding boxes are applied to the character set of textual regions, as shown in Figure 5.

The grouped keywords from localized textual regions are further processed for recognition purposes. The cropped word images $I\in {\mathbb{R}}^{W\times H}$ having width W and height H consisting of the textual cues are recognized individually. The inputs are the 2D maps resulting in a W×H map for the individual character supposition.

Given the metrics X,Y,Z and the confidence score for individual word supposition $w=(c_{1},c_{2},c_{3},\ldots ,c_{L}{}_{{}_{w}})$, let $b^{w}=(b_{1}^{w},b_{2}^{w},b_{3}^{w},\ldots ,b_{L_{w}+1}^{w})$ represent the breakpoints amid individual characters, where $b_{1}^{w}$ initializes the first character and $b_{L_{w}}^{w}$ ends the last character. The breakpoint hypothesis $\left(w,b^{w}\right)$ for the word confidence score is given as:

(22) $$s(w,b^{w},X,Y,Z)=\frac{1}{\left|b^{w}\right|}\left(\sum_{i=1}^{\left|b^{w}\right|}m_{i}(b_{i}^{w},R)+\sum_{i=2}^{\left|b^{w}\right|}\phi (b_{i}^{w},b_{i-1}^{w},X,Y,Z)\right)$$

Each individual hypothesis word w is optimized for breakpoints, and a word having an optimal score is recognized as:

(23) $$s(w,I)=\max_{b^{w}}s(w,b^{w},X,Y,Z)$$

The unary fraction scores given in Equation (22) are determined with the following properties: the distance from outside the image boundaries, the distance from the estimated breakpoint location, the binary fraction score, the non-text class score, and the distance of the first and last breakpoints from the edge of the image. The pairwise score given in Equation (23) is determined with the following properties: non-text scores at character centers, character scores at midpoints amid breakpoints, eccentricity from the normalized character width, and active contributions of the left and right binary responses comparative to character scores.

**Figure 6:**
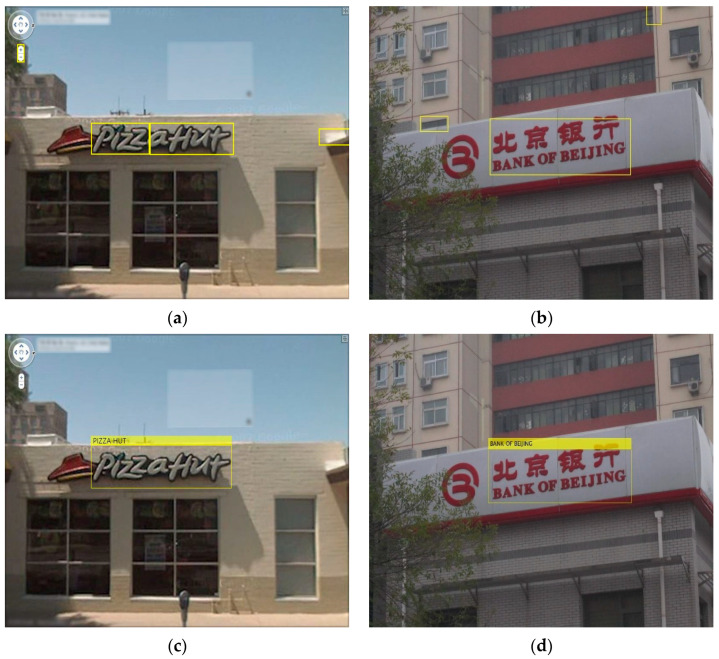
Recognized keywords in the outer environment. (**a**,**b**) Optimum bounding boxes. (**c**,**d**) Recognized keywords.

The bounding boxes are applied to recognized words in order to match the evaluated breakpoints, and the recognized bounding boxes are added to the queue of recognized words. The recognized keywords are shown in Figure 6.

### 2.2. Textual Cues Keywords

The localized textual regions are optimized with the OCR and the formal words are recognized, thus providing a sensible meaning. In this step, we utilize the recognized formal words to establish a words model that will be responsible for sequences and boundaries. Since the recognized textual cues may still be missing some characters and may affect finding the desired locations, we employ an n-gram probabilistic language model that will provide evidence for the presence of the actual cues [40].

An n-gram model is generally used to predict the probability of a given n-gram in any contiguous sequence of words. A better n-gram model predicts the next word in a sentence. For example, given the word ‘park’, the first recognized trigram is ‘par’ and the second recognized trigram is ‘ark’, and then its overlapping characters ‘ar’ suggests that the correctly recognized word is likely to be ‘park’.

Given the word w of length N as a sequence of characters $w=(c_{1},c_{2},c_{3},\ldots ,c_{N})$ where each $c_{i}\in C=\{1,2,3,\ldots ,36\}$ denotes a character at i position in word w from 26 letters and 10 digits, each recognized word has a varying length N that can be determined at the run time. Therefore, the number of characters in a single word is fixed to 22 with a null character and a maximum length class, which is given as:

(24) $$c_{i}^{*}=\arg\max_{c_{i}\in C\cup \{\phi \}}P(c_{i}|\vartheta (x))\cdot P(c_{i}|\vartheta (x))$$

For two strings and $w\in C^{M}$, the s⊂w represents s as a substring of the word w. An N-gram of w is assumed as substring s⊂w having the length |w|=N. The dictionary of all grams of word w of length N is given as:

(25) $$G_{N}(w)=\{s:s\subset w\wedge \left|s\right|\leq N\}$$

(26) $$G_{N}=\cup_{w\in \omega }G_{N(w)}$$

As an example, the dictionary for the word ‘cafe’ is $G_{3}(\mathrm{cafe})=\{c,a,f,e,\mathrm{ca},\mathrm{af},\mathrm{fe},\mathrm{caf},\mathrm{afe}\}$. Given the recognized *i*th n-gram $w_{n,i}$ and its consistent confidence score $c_{n,i}$, in order to determine the sequence of n-grams with the most confident prediction for the entire sequence of recognized words, the objective function can be given as:

(27) $$C_{n,i}=\left\{ \begin{array}{l} c_{n,i},\;\mathrm{if}\;n=1\\ \min(c_{n,i},W_{n,i}),\;\mathrm{otherwise} \end{array}\right.$$

where

(28) $$W_{n,i}=\min_{m\in [1,n-1]}\{\frac{(n-m)\cdot C_{n-m,i}+m\cdot C_{m,n+i-m}}{n}\}$$

Here, $W_{n,i}$ is used to achieve the optimal n-gram separation of the given word, and each n-gram word image is recursively recognized.

### 2.3. Similarity Learning

Similarity learning finds and matches similar images as the user-input keywords [41,42,43]. The proposed intelligent system matches the user-input keywords with the outer environment textual cues. For this, we create a feature vector of user input keywords and the recognized textual cues from the outer environment images. 

Given the input keywords Q, the word $q_{i}$ is treated as a sequence of characters $\left(y_{1},y_{2},y_{3},\ldots ,y_{\left|q_{i}\right|}\right)$, where $\left|q_{i}\right|$ denotes the total number of characters in word $q_{i}$, and $y_{j}$ is considered as the optimal representation of the $j^{th}$ character of word $q_{i}$. Each sequence is interpolated and concatenated with a fixed-length feature ${\hat{f}}_{i}\in {\mathbb{R}}^{T\times 2C}$ and all the features ${\left\{{\hat{f}}_{i}\right\}}_{i=1}^{N}$ are signified as output features $\hat{F}\in {\mathbb{R}}^{N\times T\times 2C}$.

The recognized textual cue proposals $E\in {\mathbb{R}}^{K\times T\times C}$ and the input keywords $F\in {\mathbb{R}}^{N\times T\times C}$ are formed, and the similarity is computed as a similarity matrix $\hat{S}(Q,P)\in {\mathbb{R}}^{N\times K}$ between the input keywords Q and recognized textual cues. The score ${\hat{S}}_{i,j}(Q,P)$ between both the feature vectors $F_{i}$ and $E_{j}$ is given as:

(29) $${\hat{S}}_{i,j}(Q,P)=\frac{\tanh(V(F_{i}))\tanh{\left(V(E_{j})\right)}^{T}}{\left\|\tanh(V(F_{i}))\right\|\times \left\|\tanh(V(E_{j}))\right\|}$$

where V represents the operator that converts the 2D matrix into a 1D vector. The required similarity matrix $\hat{S}(Q,P)$ is maintained by the target similarity matrix S(Q,P). The target similarity ${\hat{S}}_{i,j}(Q,P)$ is computed as the Levenshtein distance between corresponding textual pairs $\left(q_{i},q_{j}\right)$ and is given as:

(30) $$S_{i,j}(Q,P)=1-\frac{Distance(q_{i},q_{j})}{\max(\left|q_{i}\right|,\left|q_{j}\right|)}$$

Meanwhile, during implementation for the ranking, the similarity between the input keywords and recognized textual words equals to the maximum value of ${\hat{S}}_{i,j}(Q,P)$.

## 3. Experimental Results and Discussion

This section presents evaluation results to show the efficiency and accuracy of the proposed intelligent system. The evaluation protocols and benchmark datasets used for experimentation are given as follows.

### 3.1. Datasets

As the proposed intelligent system is capable to find and locate the desired locations using textual cues, we evaluated our system on four different benchmark datasets comprising the outer environment images. In these datasets, different textual cues can be found and located for the autonomous vehicle’s driving assistance system.

Street View Text (SVT): The dataset [44] consists of outer environment images and certain textual cues appear on the different objects such as walls, shops, billboards, buildings, etc. This dataset contains 250 trained images and 100 test images, and each image has a varying dimension from 1024 × 768 to 1920 × 906.

ICDAR 2013: The dataset [45] comprising the outer environment images and textual cues can be found on multiple different objects such as shops, cafes, signboards, banners, posters, etc. This dataset contains 229 trained images and 233 test images. The dimension of each image varies from 3888 × 2592 to 350 × 200.

Total-Text: The dataset [46] contains curved, orientated, and horizontal textual cues that are very challenging to detect and recognize. The text appears on multiple objects present in the outer environment. The dataset contains 300 test images and 1255 trained images and each image’s dimension varies from 180 × 240 to 5184 × 3456.

MSRA-TD500: The dataset [47] contains challenging outer and inner environment images in which the textual cues appear on doorplates, caution plates, signs, boards, etc. This dataset contains 300 trained images and 200 test images. The dimension of each image varies from 1920 × 1280 to 1296 × 864.

### 3.2. Evaluation Measures

The evaluation measures for the proposed intelligent system are given as follows.

#### 3.2.1. Textual Cues Evaluation

The detection and localization of the textual cues is one of the main entities for a robust intelligent system. We evaluate the textual cues’ detection and localization with standard evaluation protocols [48]—precision *p*, recall *r*, and frequency *f* measures defined as:

(31) $$p=\frac{\Sigma_{r_{e}\in E}m(r_{e},T)}{\left|E\right|}$$

(32) $$r=\frac{\Sigma_{r_{t}\in Τ}m(r_{t},E)}{\left|T\right|}$$

(33) $$f=\frac{1}{\frac{\alpha }{p}+\frac{\left(1-\alpha \right)}{r}}$$

#### 3.2.2. Location Retrieval Evaluation

The user-input keywords are matched with the recognized textual cues and similar location images are retrieved for the necessary actions such as applying the brake. For location retrieval, the mean average precision *mAP* is a commonly used evaluation measure which is the average of all queries. The mean average precision can be given as follows:

(34) $$P(R_{k})=\frac{\#(A\cap B)}{\#(B)}$$

where *A* denotes the number of relevant locations, *B* is the number of retrieved locations, $R_{k}$ denotes the top similar images consisting of the same textual cues as the user-specified keywords. Given the set of user keywords $q_{i}\in Q$ as $\left\{w_{1},w_{2},w_{3},\ldots ,w_{m}\right\}$, where *Q* denotes the set of all the keywords specified by the user, the *mAP* for the proposed system is formulated as:

(35) $$mAP(Q)=\frac{1}{Q}\sum_{i=1}^{\left|Q\right|}\frac{1}{m}\sum_{k=1}^{m}P(R_{k})$$

where *k* is the number of retrieved location images having the most similar textual cues.

### 3.3. Implementation Results

In this section, we briefly describe the implementation details and discuss the output results. Firstly, the proposed intelligent system asks the driver to input the keywords of the desired location. For this purpose, we randomly select the different keywords as input from the test images of all the datasets defined in Section 3.1. Secondly, the proposed system detects and localizes the textual cues from the trained images of each dataset and applies OCR recognition. Thirdly, similarity learning is applied to compute the similarity between the input keywords and recognized textual cues. Lastly, whenever any similar textual cue is found, the intelligent system informs the driver, slows down, and applies the brake to stop. The detailed experiments are given as follows.

#### 3.3.1. Textual Cues Detection

Since textual cues detection is one of the key phases for a robust intelligent system, we first evaluate the textual cues detection on the benchmark datasets. 

In this experiment, we evaluate the efficiency and accuracy of the proposed intelligent system for detecting and localizing the textual cues on the different datasets. We use the textual evaluation protocols: precision *p*, recall *r*, and frequency *f*. The obtained results of the proposed system are given and compared with the state-of-the-art methods in Table 1, Table 2, Table 3 and Table 4 for SVT, ICDAR’13, Total-Text, and MSRA-TD500 datasets, respectively.

The proposed method outperformed state-of-the-art methods for the SVT and ICDAR datasets in textual cues detection and localization. For the Total-Text dataset, our proposed method achieved better precision as compared to state-of-the-art methods. For the MSRA-TD500 dataset, the proposed method achieved remarkable results with a better *f* score. Our main target is to detect the textual cues from the low-quality road and street images such as the SVT dataset that are really challenging to detect.

#### 3.3.2. Locations Retrieval

This section describes the experimentations on the datasets for finding the desired locations. In order to show the better performance and accuracy of the proposed intelligent system, we conducted the experimentation on one-to-one and one-to-many location frames. The details of the experiments are given as follows:

**Experiment** **1.** *One-to-one: In this experiment, the proposed intelligent system first asks the driver to input the keywords of desired locations to trace and proceed. Once the driver inputs one or more keywords, the proposed system creates a feature vector of those keywords and finds a similar location image with the same textual cues. In this experiment, the proposed system keeps finding similar textual cues and asks the driver to confirm. The feature vector for input keywords continually matches with each image and produces the score. The retrieval time for this experiment is much faster than Experiment 2 as it rapidly compares the similarity and presents the outcomes to the driver. The obtained results of Experiment 1 are given in Table 5 for SVT, ICDAR’13, Total-Text, and MSRA-TD500 datasets.*

**Experiment** **2.** *One-to-many: In order to show the efficiency and accuracy of the proposed intelligent system, we perform the one-to-many experiment. In this experiment, the proposed intelligent system takes input keywords from the driver for the desired location and traces the top-rank location images possessing similar textual cues. The actual purpose of this experiment is to show the robustness of the proposed system by retrieving the top ten similar images with similar textual cues. The obtained results of Experiment 2 are compared with the state-of-the-art methods in Table 6 for SVT, ICDAR’13, Total-Text, and MSRA-TD500 datasets.*

It is worth mentioning that the proposed method outperforms the majority of the previous methods in the one-to-many experiment. However, for the ICDAR’13 dataset, our proposed method could not compete for the ICDAR’13 dataset with the method [51] but still presented a remarkable performance with other datasets.

#### 3.3.3. Retrieval Time Comparison

The time consumed during the computation of textual cues and finding the similarity is truly a critical parameter to be considered. The proposed intelligent system finds the textual features and discards the non-textual features during the localization step. The system maintains a good balance between textual and non-textual features. The retrieval time of the proposed system is compared for both experiments in Table 7. For the one-to-one experiment, the retrieval time is better and more robust since the similarity is compared between the two entities, i.e., driver-input keywords and targeted outer environment location image. For the one-to-many experiment, the retrieval time is higher since the similarity is computed to index top-rank location images.

### 3.4. Results Impact and Discussion

In this section, we discuss and compare the results of the proposed approach with state-of-the-art methods. The details are given as follows.

Textual cues: Since the textual cue detection plays a vital role in order to find the semantic locations, we first compared the textual cue detection rate for four different datasets. The output results are given on the SVT dataset for textual cues detection and localization in Table 1. The proposed approach outperformed the other methods with respect to *p*, *r*, and *f* values. The method achieved 61.52 precision, 58.31 recall, and 59.87 *f* values. Here, the precision and recall values of the method [52] are the least inferior, having 27.0 precision, 35.0 recall, and 30.0 *f* values. For the ICDAR’13 dataset, the proposed method achieved 92.4 precision, 87.63 recall, and 89.95 *f* values. The proposed method also outperformed state-of-the-art methods for the ICDAR’13 dataset. The close competent method [54] has 92.0 precision, 84.4 recall, and 88.0 *f* values. 

For the Total-Text dataset, our proposed method achieved 84.2 precision, 86.9 recall, and 85.5 *f* values. The proposed method outperformed most of the existing methods for precision and *f* values. However, the method in [60] has a slightly higher recall value, i.e., 88.6 and the method in [59] has an 87.1 recall value. For the MSRA-TD500 dataset, the proposed method achieved 86.2 precision, 86.71 recall, and 86.45 *f* values. Here, the method in [54] has the highest precision value of 87.6. However, the proposed method outperformed state-of-the-art methods for the highest recall and *f* score.

Locations retrieval: For retrieving the semantic locations, we have evaluated the proposed method for two different experiments. For the one-to-one mode, the proposed method achieved 69.2 mAP on SVT, 74.8 mAP on ICDAR’13, 59.1 mAP on Total-text, and 63.4 mAP on MSRA-TD500 datasets. The mAP is inferior due to the textual cue localization step and can be improved if the textual cues are further improved. For the one-to-many mode, the proposed method has achieved 66.8 mAP on SVT, 75.6 mAP on ICDAR, 57.4 mAP on Total-Text, and 61.7 mAP on MSRA datasets. Here, the close competent method [49] has 63.0 mAP on SVT and 71.0 mAP on ICDAR datasets. The method in [63] has the most inferior mAP 23.0 on SVT and the method in [62] has 65.0 mAP on ICDAR datasets. The proposed method has achieved an overall better mAP for all the datasets and outperformed state-of-the-art methods. 

The proposed method has certainly a few limitations. Due to its simple feature description, it is robust and able to handle only a small number of data rather than millions of images. Another disadvantage is that our method might not work well with more complicated and complex images since it might not be able to generalize to the new data type. The proposed approach, however, is exempt from both extensive training and any expensive hardware needs. The proposed approach is often easier to adopt and can be used more quickly. As a result, the proposed method performed better than the majority of the current methods and achieved an overall greater mean average precision score. The datasets included images from outer environment scenarios; however, the system’s performance can be further improved by more complicated conditions such as low light, occlusion, or a partial visibility of textual cues.

## 4. Conclusions

In this work, a novel intelligent system is proposed to sense the textual cues available in the outer environment for finding the locations in autonomous vehicles. The proposed system first asks the driver to input the keywords of the desired locations. Next, the system proceeds with the detection and recognition of certain textual cues appearing on different objects such as billboards, shops, signboards, walls, buildings, banner, etc. Whenever the system finds a location composed of similar keywords to the driver’s input keywords, the system notifies the driver, slows down, and applies the brake to stop. The experimental results on four datasets show the robustness of the proposed intelligent system for autonomous vehicles to sense the textual cues appearing in the outer environment scenario. The proposed system has lesser computation complexity and does not require any specific hardware. The system is free from a tremendous amount of training due to its simple feature description. In the future, we intend to improve the retrieval accuracy of the proposed system. We will further improve the methodology for live tracking and perform the experimentations on real-time scenario with video frames.

## Figures and Tables

**Table 1:** Textual cues detection and localization result on SVT dataset.

Method	Precision	Recall	*f*
Unar’18 et al. [49]	54.0	51.0	52.0
Wei et al. [50]	18.2	41.2	25.2
Unar’19 et al. [51]	47.0	42.0	44.0
Yu et al. [52]	27.0	35.0	30.0
Our method	61.52	58.31	59.87

**Table 2:** Textual cues detection and localization result on ICDAR’13.

Method	Precision	Recall	*f*
Wei et al. [50]	83.5	77.2	80.2
Zhong et al. [53]	90.8	86.1	88.4
Unar’18 et al. [49]	81.0	79.0	79.0
Lyu et al. [54]	92.0	84.4	88.0
Neumann et al. [36]	82.0	71.0	76.0
Unar’19 et al. [51]	83.0	82.0	82.0
Hou et al. [55]	89.6	81.3	85.3
Our method	92.4	87.63	89.95

**Table 3:** Textual cues detection and localization result on Total-Text.

Method	Precision	Recall	*f*
Wang et al. [56]	76.2	80.9	78.5
Yuxin et al. [57]	83.9	86.9	85.4
Wang et al. [58]	75.1	81.8	78.3
Liao et al. [59]	82.5	87.1	84.7
Zhang et al. [60]	75.7	88.6	81.6
Our method	84.2	86.9	85.5

**Table 4:** Textual cues detection and localization result on MSRA-TD500.

Method	Precision	Recall	*f*
Wang et al. [56]	82.1	85.2	83.6
Zhong et al. [53]	85.5	81.3	83.3
Lyu et al. [54]	87.6	76.2	81.5
Hou et al. [55]	81.8	78.7	80.2
Shi et al. [61]	86.0	70.0	77.0
Our method	86.2	86.71	86.45

**Table 5:** One-to-one location retrieval evaluation on different datasets.

Datasets	mAP
SVT	69.2
ICDAR’13	74.8
Total-Text	59.1
MSRA-TD500	63.4

**Table 6:** One-to-many location retrieval evaluation on different datasets.

Datasets	SVT	ICDAR’13	Total-Text	MSRA-TD500
Unar’18 et al. [49]	63.0	71.0	-	-
Mishra et al. [62]	56.0	65.0	-	-
Neumann et al. [63]	23.0	-	-	-
Unar’19 et al. [51]	59.0	74.0	-	-
Our method	66.8	75.6	57.4	61.7

**Table 7:** Locations’ retrieval time (s) comparison.

Datasets	One-to-One	One-to-Many
SVT	0.433	2.644
ICDAR’13	0.391	2.309
Total-Text	0.534	3.215
MSRA-TD500	0.482	2.962

## Data Availability

Data sharing is not applicable to this article.
